# Meclizine Inhibits Pseudorabies Virus Replication by Interfering With Virus Entry and Release

**DOI:** 10.3389/fmicb.2021.795593

**Published:** 2021-12-22

**Authors:** Panrao Liu, Danhe Hu, Lili Yuan, Zhengmin Lian, Xiaohui Yao, Zhenbang Zhu, Norbert Nowotny, Yi Shi, Xiangdong Li

**Affiliations:** ^1^Jiangsu Co-innovation Center for Prevention and Control of Important Animal Infectious Diseases and Zoonoses, College of Veterinary Medicine, Yangzhou University, Yangzhou, China; ^2^Viral Zoonoses, Emerging and Vector-Borne Infections Group, Institute of Virology, University of Veterinary Medicine Vienna, Vienna, Austria; ^3^CAS Key Laboratory of Pathogenic Microbiology and Immunology, Institute of Microbiology, Chinese Academy of Sciences, Beijing, China; ^4^Savaid Medical School, University of Chinese Academy of Sciences, Beijing, China; ^5^Joint International Research Laboratory of Agriculture and Agri-Product Safety, The Ministry of Education of China, Yangzhou University, Yangzhou, China

**Keywords:** pseudorabies virus, meclizine, antiviral activity, virus entry, virus release

## Abstract

Pseudorabies virus (PRV) is a pathogen that causes substantial economic losses to the swine industry. With the emergence and widespread of PRV variants since 2011 in China, current commercial vaccines cannot provide complete protection against PRV infection. Therefore, antiviral drugs may work as an alternative way to control and prevent PRV. In this study, the inhibitory effects and underlying molecular mechanisms of meclizine against PRV were studied. Meclizine displayed a significant inhibitory effect against PRV when it was added before, simultaneously with, or after virus infection. The inhibitory effect of meclizine occurred during viral entry and cell-to-cell spreading but not at viral attachment into PK-15 cells. Meclizine also inhibited viral particle release at the late stage of infection. The antiviral effect of meclizine was tested in mice, and the results showed that meclizine reduced the severity of clinical symptoms and the viral loads in tissues, and delayed the death, after PRV challenge. The above results indicated that meclizine had an inhibitory effect on PRV. Our findings will contribute to the development of potential therapeutic drugs against PRV infection.

## Introduction

Pseudorabies (PR) is an acute highly contagious disease caused by pseudorabies virus (PRV). It was first described in Hungary in 1902 (Lee and Wilson, 1979). PRV, an enveloped and double-stranded linear DNA virus, is a member of the *Herpesviridae* family (Sun et al., 2016). It was also called Aujeszky’s disease virus (ADV), which causes fever, itching (except pigs), and encephalomyelitis in many livestock and wild animals (Müller et al., 2011; Tan et al., 2021). PRV can infect pigs of different ages. The clinical signs of infected pigs were fever, diarrhea, vomiting, nervous system disorders, with high mortality for newborn piglets, nonsignificant symptoms for adult pigs, abortion, stillbirth and respiratory symptoms for sows, and reproductive disorder for boars (Tan et al., 2021). Recently, it has been reported that PRV infects and causes human endophthalmitis or encephalitis, which highlights the potential threat of this pathogen to public health (Wong et al., 2019; Yang et al., 2019; Liu et al., 2020).

In China, PRV were firstly identified in cats in 1947, then were reported in swine and other animals. At present, both inactivated and attenuated live vaccines are widely used to prevent and control PR (Freuling et al., 2017; Delva et al., 2020). However, PRV variants emerged and spread in China since 2011, leading to huge economic losses (An et al., 2013; Wu et al., 2013; Tong et al., 2015). Compared to classical virulent PRV, PRV variants were more pathogenic on pigs and commercial vaccines failed to provide complete protection against the variants (Luo et al., 2014; Hu et al., 2021).

Besides developing new vaccines using current PRV circulating strains, researchers have also been working to identify inhibitors or drugs against PRV infection. Some diaminopurine-based acyclic nucleoside phosphonate analogues exhibited effective anti-PRV activity (Zouharova et al., 2016). Resveratrol, a polyphenolic stilbenoid, was identified to show efficient anti-PRV activities *in vitro* and *in vivo* (Zhao et al., 2017, 2018). The inhibitory effect of resveratrol occurred during viral multiplication by inhibiting IκB kinase activity but not at viral entry into porcine kidney cells (PK-15; Zhao et al., 2017). Further studies showed that resveratrol treatment effectively relieved pathological symptoms, reduced PRV-induced inflammation, and increased the growth performance of PRV-infected piglets (Zhao et al., 2018). Fang et al. (2020) demonstrated that hydroquinone inhibited PRV replication during viral attachment and internalization into PK-15 cells by activating the phosphorylation of AKT. In addition, platycodon grandiflorus polysaccharides were confirmed to inhibit PRV replication *via* downregulating PRV-induced autophagy (Xing et al., 2021).

Meclizine is a first-generation piperazine class of H1-antihistamine, which are usually used for vertigo, nausea and vomiting (Cohen and Dejong, 1972). Meclizine can also treat the same symptoms caused by viral infection, pregnancy, or radiation therapy. Meclizine has some anticholinergic activity as H1-antihistamines, and it is also reported to regulate constitutive androstane receptor (Huang et al., 2004). Recently, meclizine has been identified as an inhibitor of mitochondrial respiration or mitochondrial oxidative phosphorylation (Gohil et al., 2010, 2013). Gohil et al. revealed that meclizine inhibited mitochondrial respiration by inhibiting phosphoethanolamine cytidylyltransferase (PCYT2) activity. HSV-1 replication was significantly reduced after treatment with meclizine in Hela cells and in mice experiment (Arii et al., 2020).

In this study, we investigated whether meclizine could inhibit PRV replication *in vitro* and *in vivo*. Our findings showed that meclizine displayed antiviral activity against PRV infection in PK-15 cells, while the antiviral effect was not significant in mice. The mechanisms of meclizine still need to be further clarified. The study laid a foundation for the development of potential therapeutic drugs against PRV infection.

## Materials and Methods

### Ethics Statement

All the animal experiments were approved by the Jiangsu Administrative Committee for Laboratory Animals (Permission Number: SYXKSU-2017-0044) and complied with the Guidelines of Laboratory Animal Welfare and Ethics of Jiangsu Administrative Committee and Laboratory Animal Welfare and Ethics Committee of Yangzhou University for Laboratory Animals.

### Cells and Virus

PK-15 cells were obtained from the American Type Culture Collection (ATCC) and cultured in Dulbecco’s modified Eagle medium (DMEM, CORNING, United States) containing 10% fetal bovine serum (FBS, Thermo Fisher Scientific, Waltham, United States) and 1% penicillin/streptomycin at 37°C with 5% CO_2_. PRV variant strain JS21 with abbreviation of PRV-W and PRV Bartha K61 strain (GenBank accession no. JF797217; with abbreviation of PRV-V) were preserved in our laboratory. PRV titers were determined as the median tissue culture infective doses (TCID_50_) on PK-15 cells.

### Cell Viability Assay

The viability of PK-15 cells after meclizine treatment was determined using the Enhanced Cell Counting Kit-8 (CCK-8; Beyotime, Shanghai, China) to detect the relative cytotoxicity of agents according to the manufacturer’s instructions. Briefly, PK-15 cells were seeded in 96-well plates (1 × 10^4^ cells/well) and then exposed to different concentrations of meclizine or DMSO. Plates were incubated for 24 h at 37°C with 5% CO_2_. After incubation, added 10 μl CCK-8 solution (containing WST-8, which can be reduced by some dehydrogenase in mitochondria to form orange formazan) to each well and continue incubated for 3 h. The absorbance was measured at 450 nm.

### Virus Infection

PK-15 cells were seeded into 6-well plates (1 × 10^6^/well) overnight at 37°C with 5% CO_2_. When the cells reached approximately 70–80% confluence, they were infected with PRV at a multiplicity of infection (MOI) of 1 and incubated at 37°C with 5% CO_2_ for 1 h. Then the supernatant was removed by washing with phosphate-buffered saline (PBS) for three times and incubated in DMEM supplemented with 2% FBS.

### Meclizine Treatment

PK-15 cells were seeded into 6-well plates (1 × 10^6^/well) overnight at 37°C with 5% CO_2_. When the cells reached 70–80% confluence, they were infected with PRV (PRV-W and PRV-V) at MOI of 1 and cells were treated with meclizine at different concentrations (50, 100, 150, and 200 μM) or DMSO (used as negative control). Twenty-four hours later, the supernatant and cell samples were harvested for further detection.

### Western Blot Analysis

PK-15 cells seeded in 6-well plates were washed with cold PBS and harvested with lysis buffer (containing 50 mM Tris (pH 7.4), 150 mM NaCl, 1% Triton X-100, 1% sodium deoxycholate, 0.1% SDS, etc.; Beyotime, Shanghai, China) on ice. After centrifugation, the supernatant was denatured and subjected to SDS-PAGE and transferred to polyvinylidene difluoride (PVDF) membranes (Millipore, MA, United States). The membranes were blocked in TBST with 5% nonfat dry milk for 1 h at room temperature and then incubated with antibodies of anti-PRV glycoprotein B (gB) mAb (1:1,000, preserved in our laboratory) and anti-glyceraldehyde-3-phosphate dehydrogenase (GAPDH; 1:1,000, Cell Signaling Technology, MA, United States) overnight at 4°C. After washing, the membranes were incubated with HRP-conjugated secondary antibodies for 1 h at room temperature (Jackson ImmunoResearch, PA, United States). The signals were visualized using an enhanced chemiluminescence reagent kit (NCM Biotech, Suzhou, China) through Tanon 5200 system (Tanon, Shanghai, China).

### Immunofluorescence Assay

The cells were washed with PBS and fixed with 4% paraformaldehyde for 10 min and permeabilized with 0.5% Triton X-100 at room temperature. After blocking with 3% bovine serum albumin (BSA) in TBST for 30 min, the cells were incubated with anti-PRV gB protein mAb (1:1,000) for 1 h, followed by incubation with FITC-conjugated anti-mouse IgG secondary antibody (1:500, Cell Signaling Technology, MA, United States) for 45 min at 37°C. The cell nuclei were stained with DAPI (Beyotime, Shanghai, China) for 10 min at room temperature. Finally, the cells were visualized with a LSM 880 Zeiss confocal fluorescence microscope (Oberkochen, Germany).

### DNA Extraction and Quantitative PCR

Total DNAs from cells or tissues were extracted using a DNA Extraction Kit according to the manufacturer’s instructions (Omega, GA, United States). Viral loads were tested and assayed by real-time PCR. Briefly, serial 10-fold dilutions of the PRV-gD standard plasmid were used to construct a standard curve for each experiment. The CT value of the sample is substituted into the standard curve to calculate the corresponding copy number of viral genome DNA. Primers and probes are as follows: PRV-F: GTGGGCGTG TGCGTCTACA, PRV-R: GACCGGGCTGCGCT TTTA, the probe: FAM-CGAAGGGGTATCGCCTCCT-BHQ1. The PCR Mix (TaKaRa, Dalian, China) was used following the manufacturer’s recommendations. The Quantitative PCR (qPCR) was performed on an ABI QuantStudioTM 3 (Applied Biosystems, CA, United States). The qPCR reaction was performed under the following conditions: 95°C for 1 min, followed by 40 cycles at 95°C for 5 s and 60°C for 1 min.

### Viral Attachment, Entry, Replication, and Cell-to-Cell Spreading Assays

In the viral attachment assay, PK-15 cells were pre-cooled at 4°C for 30 min and then challenged with PRV strains (MOI = 5) with or without meclizine (150 μM) for 3 h at 4°C. After washing with PBS for three times, the cells were harvested for qPCR to test and analyze the copy numbers of PRV DNA.

In the viral entry assay, PK-15 cells were infected with PRV strains (MOI =5) for 3 h at 4°C. After washing, the cells were incubated at 37°C and meclizine was added at scheduled time points. Meclizine (150 μM) was added at 0, 0.5, 1, 1.5, 2, and 4 h (the time when the cells were transferred to 37°C was set as 0 h). After incubation for 6 h, the cells were washed for three times and harvested for qPCR to quantify viral DNA.

In the viral release assay, PK-15 cells were infected with PRV strains (MOI = 0.1/0.01) at 37°C for 24 h. After washing with PBS for three times, the medium was replaced with DMEM of 2% FBS and meclizine (150 μM) for another 4 h in 5% CO_2_ at 37°C. Then the supernatants were harvested to quantify the viral DNA using qPCR.

In the cell-to-cell spreading assay, PK-15 cells were infected with PRV strains (MOI = 1) and treated with meclizine (150 μM) in 5% CO_2_ at 37°C. After washing with PBS for three times, PK-15 cells were collected for immunofluorescence assay (IFA) at 0, 6, 9, 12, 18, and 24 h post-infection.

### Animal Experiments

Twenty 5-week-old female BALB/c mice were randomly divided into five groups: control group, the administration group (Mec100), PRV-infected group (PRV), PRV-infected and administration groups (meclizine at doses of 50 mg/kg and 100 mg/kg respectively, PRV + Mec50, PRV + Mec100). Mice in different groups were fed separately. The infected mice were intraperitoneally injected with PRV-W at a dose of 10^2.5^ TCID_50_. The administration group was injected with meclizine at doses of 100 mg/kg. The control group was injected with the same amount of DMEM. The other two groups were challenged with PRV in the same way and dose. At 8 h after PRV infection, the mice were administered intraperitonially with meclizine at doses of 50 mg/kg or 100 mg/kg respectively, which was repeated every 12 h until the end of the study. Meanwhile, 0.9% NaCl solution was injected into the first three groups. After inoculation, the daily behavior, mental status, and survival of mice were monitored every day for 7 days. Mice were humanely euthanized and brain, lung, and liver samples were subjected for gross pathology examination. DNA extraction and the viral load in tissues were quantified by qPCR.

### Statistical Analysis

GraphPad Prism 7.0 (GraphPad Software, CA, United States) was used for the statistical analyses. The data was analyzed with student’s t test and one-way test among groups and expressed as the mean ± standard deviation. The values of *p* of <0.05 was considered as statistically significant. Significance in all figures is indicated as follows: *, *p* < 0.05; **, *p* < 0.01; ***, *p* < 0.001.

## Results

### Meclizine Inhibited PRV Infection in PK-15 Cells

**Figure 1:**
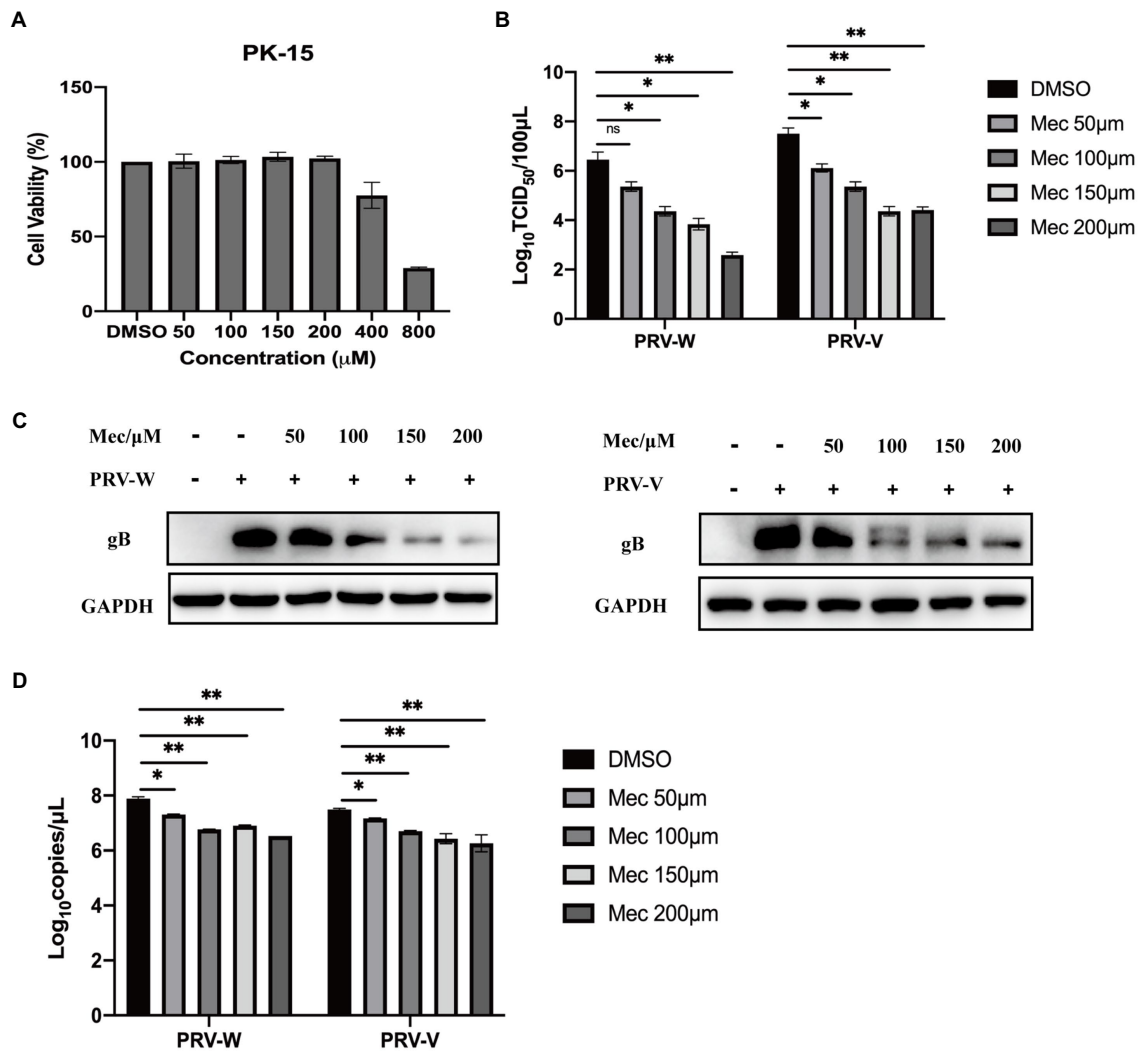
Meclizine inhibited PRV infection and replication in PK-15 cells. **(A)** Potential cytotoxicity of meclizine against PK-15 cells was detected with the Enhanced Cell Counting Kit-8. PK-15 cells were seeded in 96-well plates and then exposed to different concentrations of meclizine or DMSO. After 24 h of incubation, 10 μl CCK-8 solution was added for another 3 h. The absorbance was measured at 450 nm. **(B–D)** PK-15 cells were infected with PRV variant strain (PRV-W) and Bartha K61 strain (PRV-V) at MOI of 1 in the presence of different concentrations of meclizine, DMSO (control) for 24 h at 37°C with 5% CO_2_. The supernatant and cell samples were harvested. Supernatants were subjected to virus titration. The expression levels of gB protein were analyzed by immunoblotting **(C)**. The copy numbers of PRV-W and PRV-V DNA were quantified by real-time PCR **(D)**. Data were presented as means±SD from three independent experiments. *, *p* < 0.05; **, *p* < 0.01; ***, *p* < 0.001.

**Figure 2:**
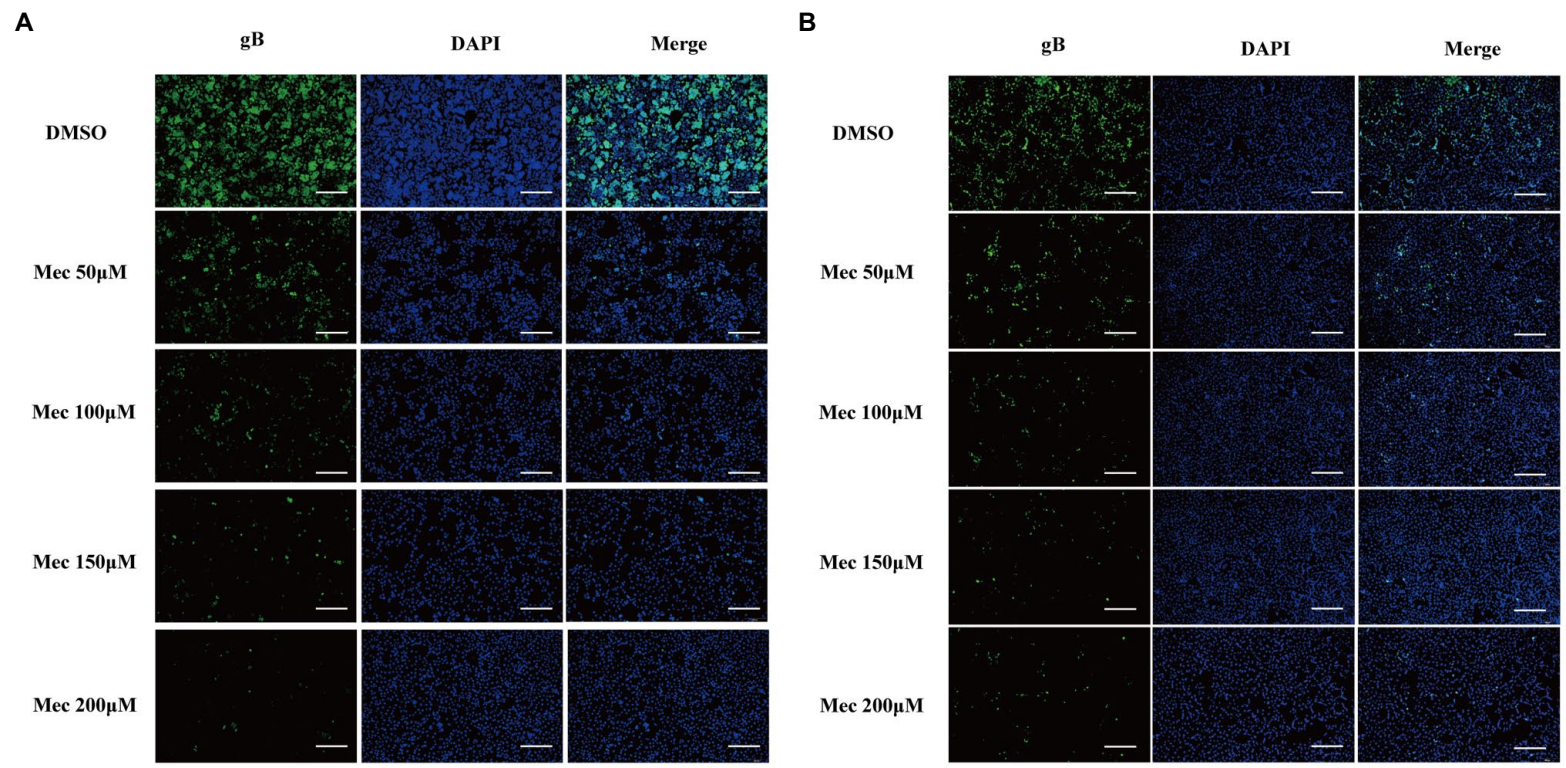
Meclizine decreased the expression levels of PRV gB protein detected by IFA. PK-15 cells were infected with PRV-W **(A)** and PRV-V **(B)** at MOI of 1 in the presence of different concentrations of meclizine or DMSO (control groups) for 24 h at 37°C with 5% CO_2_. Cells were detected by IFA. Scale bars = 200 μm. Typical figures were presented from three independent experiments.

To evaluate the antiviral activity of meclizine against PRV infection, we first investigated its cytotoxicity on PK-15 cells. The viability of PK-15 cells after different concentration of meclizine treatment for 24 h was determined. Compared with DMSO control, no significant cytotoxicity was observed when the drug concentration did not exceed 200 μM (Figure 1A). To further examine the antiviral effect of meclizine on different PRV strains, PK-15 cells were treated with meclizine of 50, 100, 150, and 200 μM and infected with PRV-W and PRV-V at a MOI of 1, respectively. At 24 h post infection (h.p.i.), virus titration, immunoblotting, qPCR, and IFA assays were performed. As shown by Figure 1B, virus titers in PK-15 cells significantly decreased by meclizine (≥100 μM) compared with the DMSO control. The results of immunoblotting showed that the expression levels of PRV-gB protein were markedly reduced by meclizine (Figure 1C). The inhibitory effect was more significant when meclizine was used in higher concentration. Besides, a notable decrease of copy numbers of PRV was also observed in meclizine-treated groups by using qPCR (Figure 1D). Consistently, the fluorescence intensity of cells treated with meclizine was significantly weaker than that in the DMSO groups (Figure 2). These results indicated that treatment with meclizine effectively inhibited PRV infection in PK-15 cells.

### Meclizine Inhibited PRV Infection During the Different Stages of Infection

**Figure 3:**
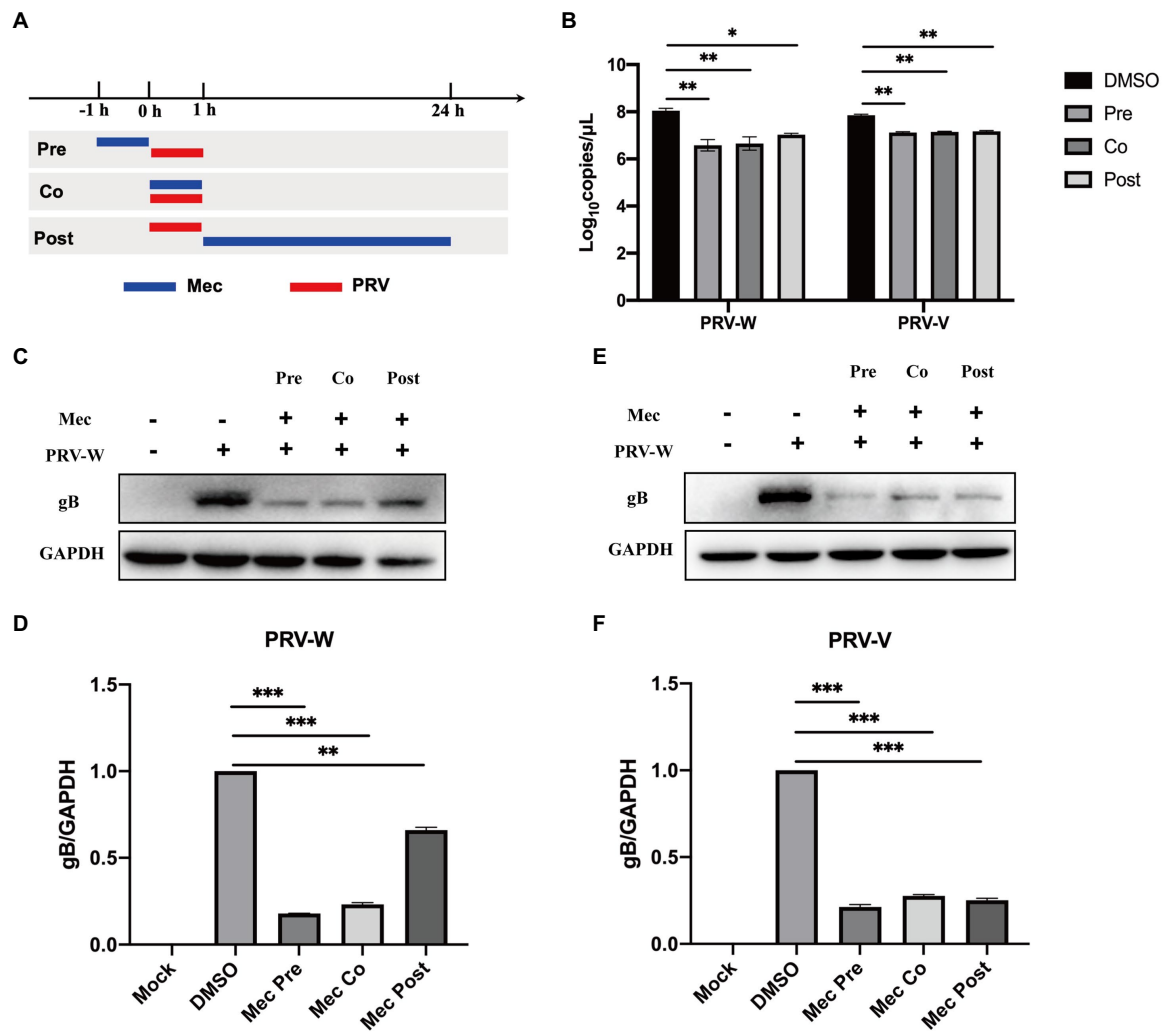
Meclizine inhibited PRV infection during the different stages of infection. **(A)** Schematic diagram of meclizine administration. PK-15 cells were infected with different PRV strains (PRV-W and PRV-V) at MOI of 1 for 1 h, and cells were treated with 150 μM meclizine at different time points. Meclizine treatment was performed either before (pre), simultaneously with (co), or after PRV infection (post), respectively. At 24 h, cell samples were harvested. **(B)** The copy numbers of PRV DNA were quantified by qPCR. **(C–F)** The expression levels of gB protein and GAPDH were analyzed by immunoblotting. The analysis of WB band gray value was visualized by Image J software. Data were presented as means±SD from three independent experiments. *, *p* < 0.05; **, *p* < 0.01; ***, *p* < 0.001.

To investigate the stages that meclizine affects PRV infection *in vitro*, meclizine treatment was performed either before, simultaneously with, or after PRV infection, respectively (Figure 3A). PK-15 cells were treated with meclizine of 150 μM and infected with different PRV strains at a MOI of 1. At 24 h.p.i., cells were harvested for qPCR and Western blot analysis. Compared with DMSO control, the production of viral DNA was significantly decreased with meclizine at three different approaches (Figure 3B). Consistent with qPCR results, the expression of gB was significantly reduced with treatment of meclizine on protein level (Figures 3C–F). In addition, the inhibitory effect was the most significantly in meclizine treatment before PRV infection. These results showed that meclizine inhibited PRV infection during the different stages of infection, particularly in the early stage.

### Meclizine Inhibited PRV Infection by Interfering With Virus Entry, Release, and Cell-to-Cell Spreading

**Figure 4:**
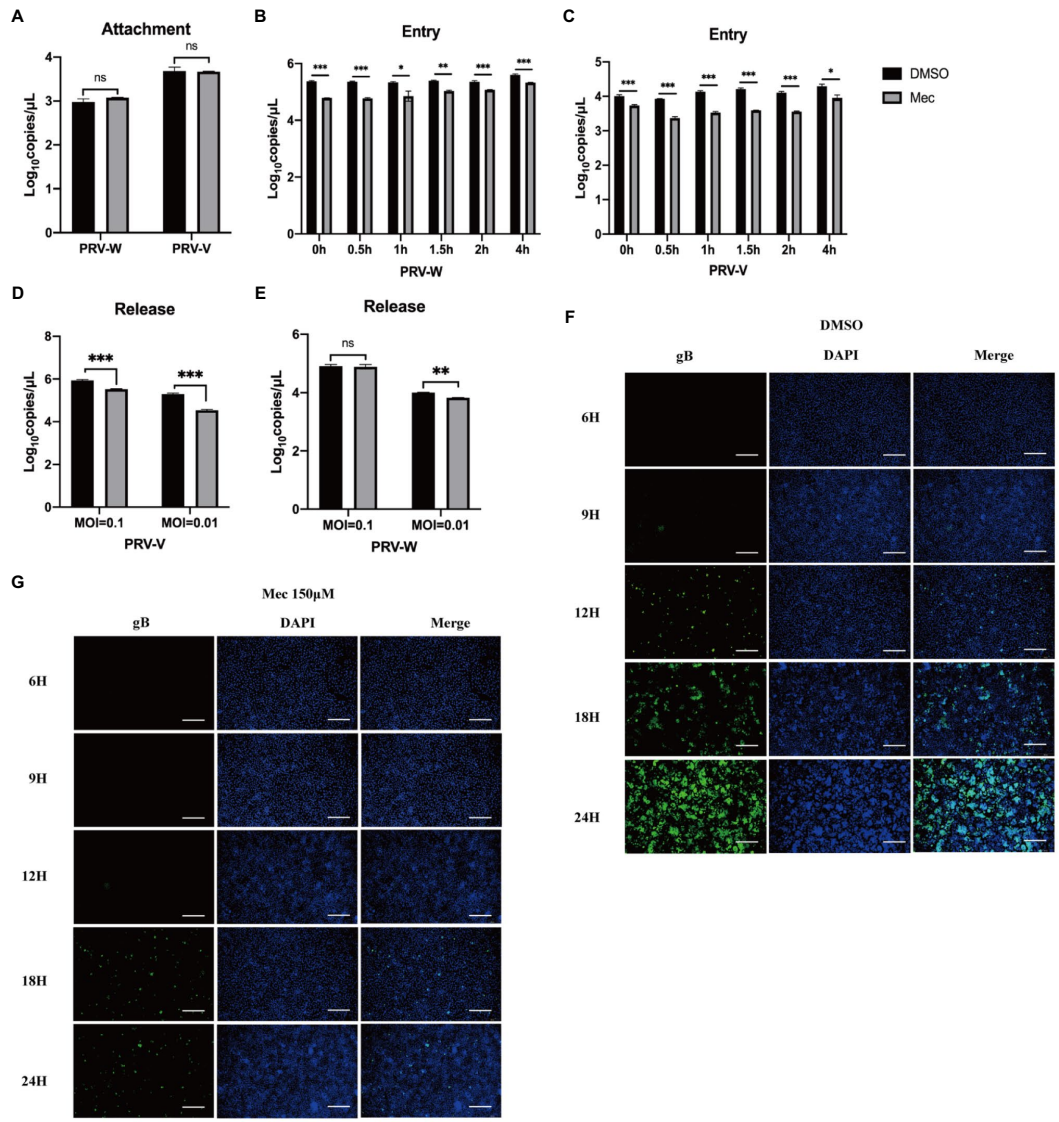
Meclizine inhibited PRV infection by interfering with the entry, release and cell-to-cell spreading but not attachment into PK-15 cells. PK-15 cells were seeded in 6-well plates and cultured overnight. When the cells reached approximately 70–80% confluence before carrying out antiviral assays. **(A)** Virus attachment assay. **(B,C)** Virus entry assay. **(D,E)** Virus release assay. **(F,G)** The cell-to-cell spreading assay of meclizine about PRV-W. The results of PRV-V were showed in Supplementary Figure S1. Scale bars = 200 μm. Data were presented as means±SD from three independent experiments. *, *p* < 0.05; **, *p* < 0.01; ***, *p* < 0.001.

Replication cycle of PRV includes four stages: adsorption, entry, replication, and release. To explore the mechanism of meclizine against PRV infection, viral attachment assay, entry assay, release assay, and cell-to-cell spreading assay were performed. For the viral attachment assay, PK-15 cells were infected with PRV with or without meclizine. After washing, the cells were harvested for qPCR to quantify the copy number of viral DNA. The results showed that the copy numbers of PRV DNA in meclizine-treated cells were not significantly different from those in the control cells (Figure 4A).

For the viral entry assay, PRV-infected cells were incubated at 37°C and meclizine was added at scheduled time points. Finally, intracellular viral DNA was quantified using qPCR. The results showed that the copy numbers of PRV DNA were remarkable decreased after adding meclizine (Figures 4B,C). Interestingly, the inhibition effect was better when meclizine was added at the earlier stage of incubation at 37°C.

For the viral release assay, the medium was replaced with DMEM containing 2% FBS and meclizine (150 μM) at 24 h after PRV infection. Four hours later, the supernatants were harvested to quantify the viral DNA using qPCR. Compared to control cells, the copy numbers of PRV DNA in meclizine-treated cells were significantly lower (Figures 4D,E).

As shown in Figure 4F, the fluorescence intensity in the DMSO groups increased over time after PRV-W infection. However, the amount of fluorescence in the cells treated with meclizine was much late and significantly less than that in DMSO groups (Figure 4G). Expectedly, meclizine also showed a similar effect during PRV-V infection, the results were showed in Supplementary Figure S1. The results indicated that meclizine inhibited cell-to-cell spreading step during PRV replication.

Taken together, these data indicated that meclizine negatively affected the entry, release, and cell-to-cell spreading stages of viral life cycle to inhibit PRV infection.

### Meclizine Inhibition on PRV Infection *in vivo*

**Figure 5:**
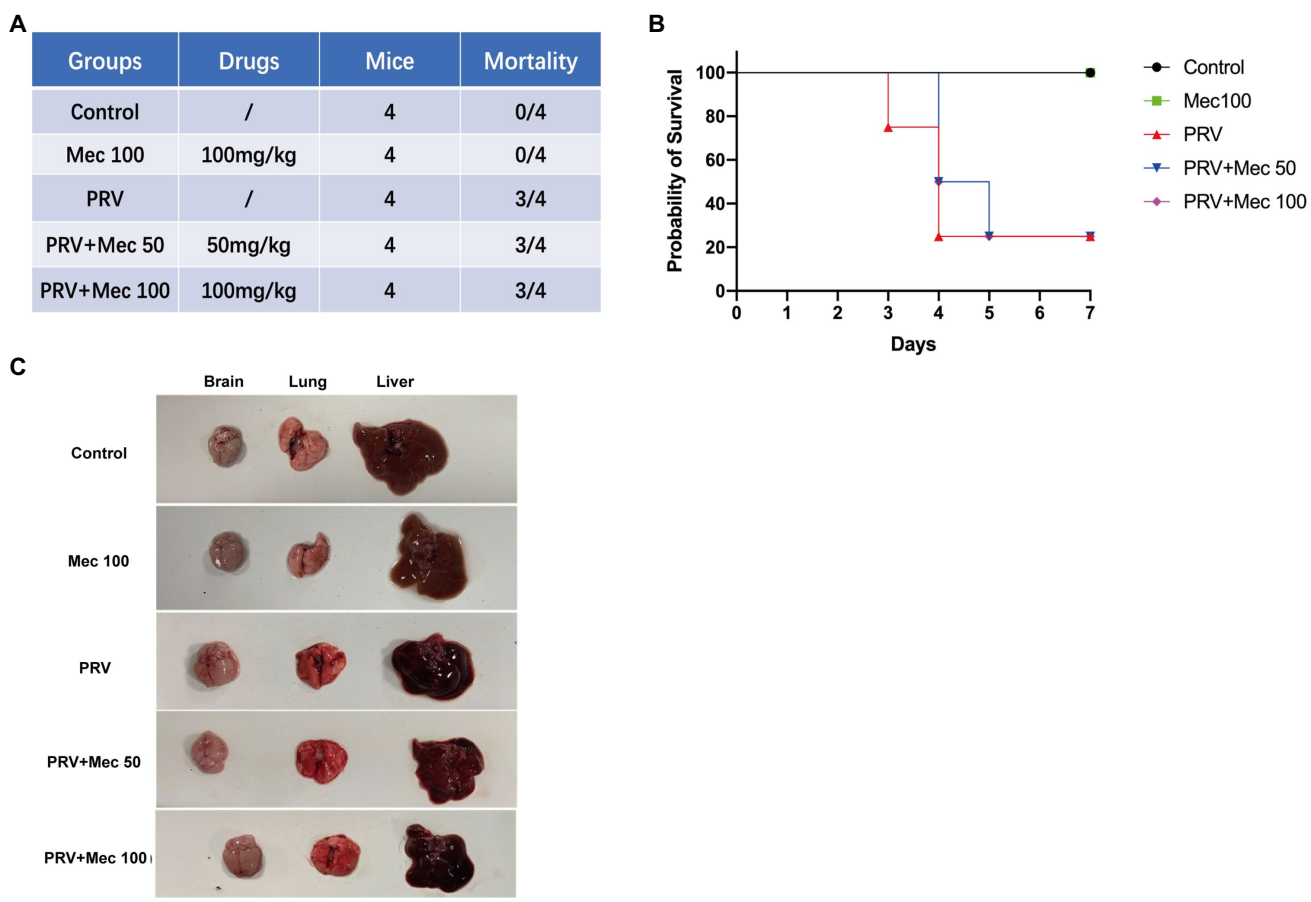
Meclizine inhibits PRV infection in mice. **(A)** Schematic diagram of mice experiment and mortality of mice in different groups. **(B)** Survival profile of mice after PRV challenge in different groups. **(C)** Brain, lung, and liver samples of mice were collected for pathological examination. Typical figures were presented from three independent experiments.

After PRV-W challenge, the daily behavior, mental status, and survival of mice were monitored and recorded. Mice that were not administered with meclizine showed clinical symptoms, including loss of appetite, lethargy, and scratching. On the 3rd day after viral challenge, the mice in the PRV group began to die and the mortality rate was 75% on the 4th day (Figures 5A,B). While the mice in PRV + Mec groups were found dead from the 4th day. At the end of the experiment, there was one mouse survived in both PRV group and PRV + Mec group. All mice in control group and administration group survived within 7 days (Figure 5B). Brain, lung, and liver samples of mice were collected and subjected to gross pathology evaluation. Pathological examination results showed that lungs and brains of mice in PRV group had hemorrhage and congestion, which were more severe than that in PRV + Mec groups. There was more serious edema in livers of mice in PRV group than in other groups (Figure 5C). No significant lesions were observed in the tissues from Mec100 group and control group.

**Figure 6:**
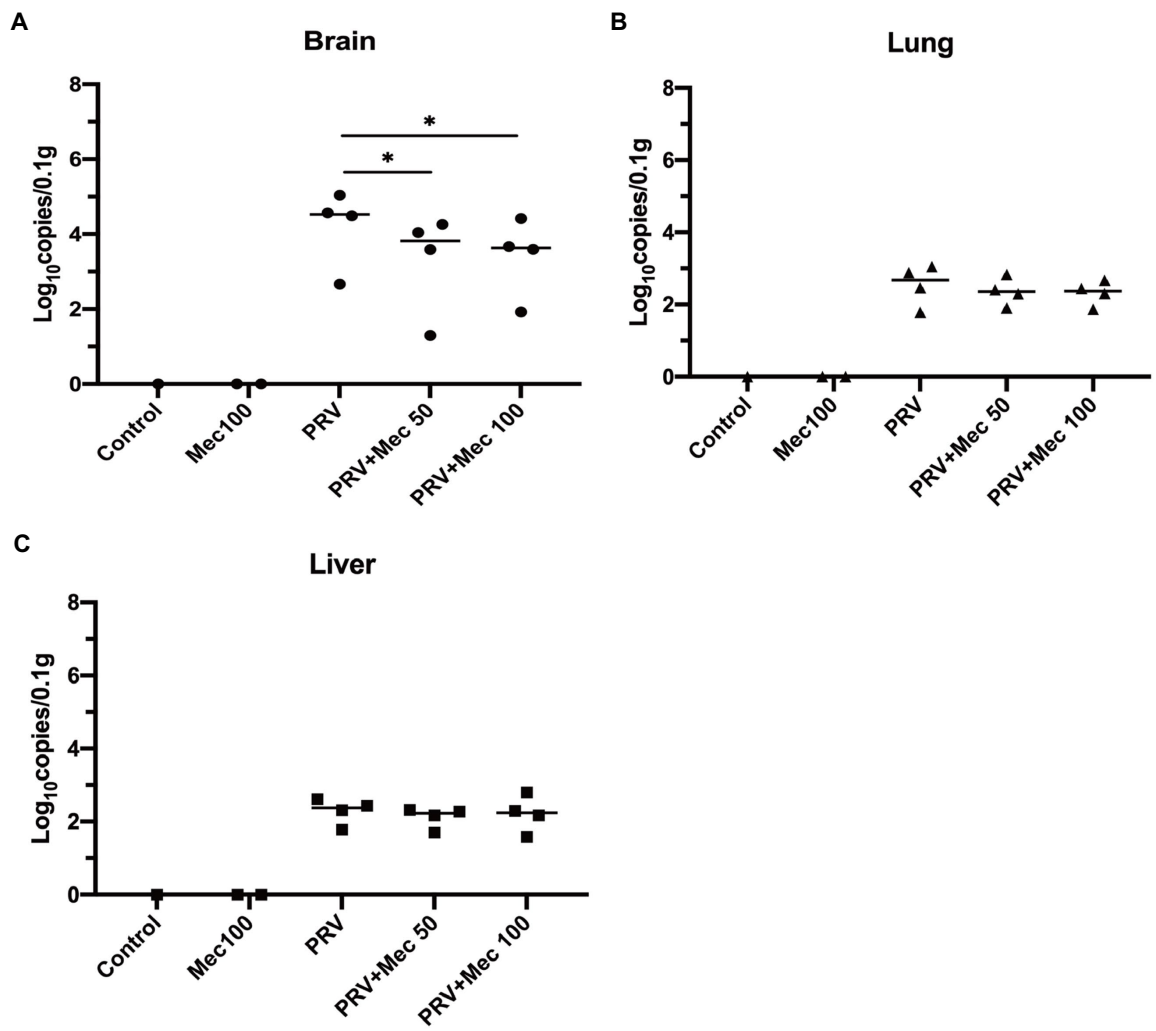
Meclizine treatment decreased the viral loads in tissues after viral challenge. 0.1 g of brain **(A)**, lung **(B)**, and liver **(C)** samples were taken for DNA extraction. The viral loads in tissues were analyzed by qPCR. *, *p* < 0.05; Data were presented as means ± SD from three independent experiments.

In addition, viral load in tissues were quantified to further explore the effect of meclizine on PRV replication *in vivo*. The results of qPCR showed that the viral loads in mice brain were higher than that in the lungs and livers (Figure 6). PRV loads in the brains of mice from PRV + Mec groups were lower than those from PRV group (Figure 6A). There was no significant difference among PRV group and PRV + Mec groups in the lungs and livers of mice (Figures 6B,C). The above results suggested that meclizine treatment could decrease the severity of clinical symptoms, delay death, and reduce viral loads in tissues after viral challenge. However, the inhibit effect of meclizine in mice were not as good as those *in vitro*, and the mechanism was unknown.

## Discussion

Since the emergence and widespread of PRV variants, commercial vaccines could not prevent completely PRV infection in pigs, which led to huge economic losses to the Chinese pig industry. Besides developing new PRV vaccines, research on antiviral drugs with inhibitory effects on PRV is an alternative solution for disease control. In this study, we for the first time verified that meclizine had potent inhibitory effect on PRV variant strain and Bartha K61 strain both *in vitro* and *in vivo*.

Our results indicated that meclizine reduced PRV replication at multiple stages of virus life cycle by impairing the production of viral particles and protein synthesis (Figure 3). Antiviral assay of meclizine administration with three different methods showed that meclizine treatment no matter before or simultaneously with PRV infection displayed more effective antiviral activity against PRV replication than treatment after PRV infection. Antiviral drugs were reported to destroy virus replication by targeting different stages of life cycle of infectious viruses, such as the attachment, entry, and release of viral particles (Yasin et al., 2004; Kausar et al., 2021; Tompa et al., 2021). As for meclizine, our results suggested it could interfere with viral entry, release and cell-to-cell spreading stages of the viral life cycle (Figure 4), which was similar to the effect of it inhibiting HSV-1 replication (Arii et al., 2020).

Viruses interact and regulate with cell membrane in several stages of replication, which need to pass through cell membrane to entry for infection and exit for release virus particles (Lorizate and Kräusslich, 2011). Lipids are the structural basis of cell biofilm, which mainly include glycerophospholipids, sphingolipids, and sterols. Glycerol phospholipids in cell biofilms and viral envelope play a variety of roles in membrane related virological events (Ketter and Randall, 2019). Phosphatidylethanolamine (PE) is a kind of glycerophospholipids, which is involved in the composition of biofilm (Hishikawa et al., 2014). Meclizine is also an inhibitor of metabolic enzyme PCYT2, in addition to being an antihistamine. PCYT2 is a key enzyme for the biosynthesis of PE from ethanolamine and diacylglycerol (Pavlovic and Bakovic, 2013; Vance and Tasseva, 2013). In our study, PRV replication was significantly decreased after treatment with meclizine. The possible mechanism could be attributed to PE biosynthesis pathway was disrupted by meclizine and reduced the production of cell biofilms, which were adverse to virus entry, protein biosynthesis, and virus egress. The antiviral mechanism of meclizine needs further investigation.

The antiviral activity of meclizine was also tested in mice. Compared with PRV group, the severity of clinical symptoms was decreased, and death of mice in the PRV + Mec groups was delayed (Figure 5). Decreased viral loads were observed in tissues of mice in PRV + Mec groups compared with those in PRV group (Figure 6). The viral loads in the brain were higher than that in the lungs and livers, which was consistent with the previous research (Huang et al., 2020). In our study, meclizine only delayed the death of mice but not reduce the mortality. It suggested that the inhibitory mechanism of meclizine on PRV was complex, and its effect in mice may be inconsistent with those *in vitro*. In addition to pharmacological activity, the function of meclizine in mice was also related to their absorption, distribution, metabolism, excretion and so on. When drugs act on cells, they can exert their effect directly. When the drug is used in animals, it needs to enter the blood circulation through the barrier membrane (vascular wall and mucosa, etc.) from the site of administration, then be distributed in various organs or cells, and be absorbed by the body to play roles. Meanwhile, with the prolongation of time, the drug will undergo varying degrees of structural changes and gradually be metabolized by the body, which led to its pharmacological effects weakened or completely lost. In our experiment, although mice were administered with meclizine every 12 h, the time to maintain effective working concentration of meclizine was still unclear. It was possible that medicine had a short time to maintain its antiviral activity in mice and did not display the expected protective effect on them. The detailed mechanism was unknown.

To sum up, our findings revealed that meclizine not only has antiviral activity, but also showed inhibitory effects on PRV replication *in vitro*, when it was administered either before, simultaneously with, or after PRV infection, respectively. Meclizine also decreased viral replication in brains *in vivo*. Therefore, meclizine has the potential for the development of preventive and therapeutic strategies for PRV infection.

## Data Availability Statement

The original contributions presented in the study are included in the article/Supplementary Material; further inquiries can be directed to the corresponding authors.

## Ethics Statement

The animal study was reviewed and approved by the Laboratory Animal Welfare and Ethics Committee of Yangzhou University.

## Author Contributions

PL, YS, and XL conceived and designed the experiments. PL and DH performed the experiments, analyzed the data, and drafted the manuscript. LY, ZL, XY, and ZZ contributed reagents, materials, and analysis tools. PL, NN, YS, and XL thoroughly revised the manuscript. All authors contributed to the article and approved the submitted version.

## Funding

This work was funded by the National Natural Science Foundation of China (nos. 32102637 and 32172823), Yangzhou University Interdisciplinary Research Foundation for Veterinary Medicine Discipline of Targeted Support (to YS), and the Project of the Priority Academic Program Development of Jiangsu Higher Education Institutions (PAPD).

## Conflict of Interest

The authors declare that the research was conducted in the absence of any commercial or financial relationships that could be construed as a potential conflict of interest.

## Publisher’s Note

All claims expressed in this article are solely those of the authors and do not necessarily represent those of their affiliated organizations, or those of the publisher, the editors and the reviewers. Any product that may be evaluated in this article, or claim that may be made by its manufacturer, is not guaranteed or endorsed by the publisher.

## Supplementary Material

The Supplementary Material for this article can be found online at: https://www.frontiersin.org/articles/10.3389/fmicb. 2021.795593/full#supplementary-material

Supplementary Figure S1 Meclizine inhibited cell-to-cell spreading step during PRV-V replication. Scale bars = 200 μm. Typical figures were presented from three independent experiments.
