# Supplementary figures and images for: Meclizine Inhibits Pseudorabies Virus Replication by Interfering With Virus Entry and Release
Source: Front Microbiol. 2021 Dec 22;12:795593. doi: 10.3389/fmicb.2021.795593 (PMC8727530; doi:10.3389/fmicb.2021.795593)

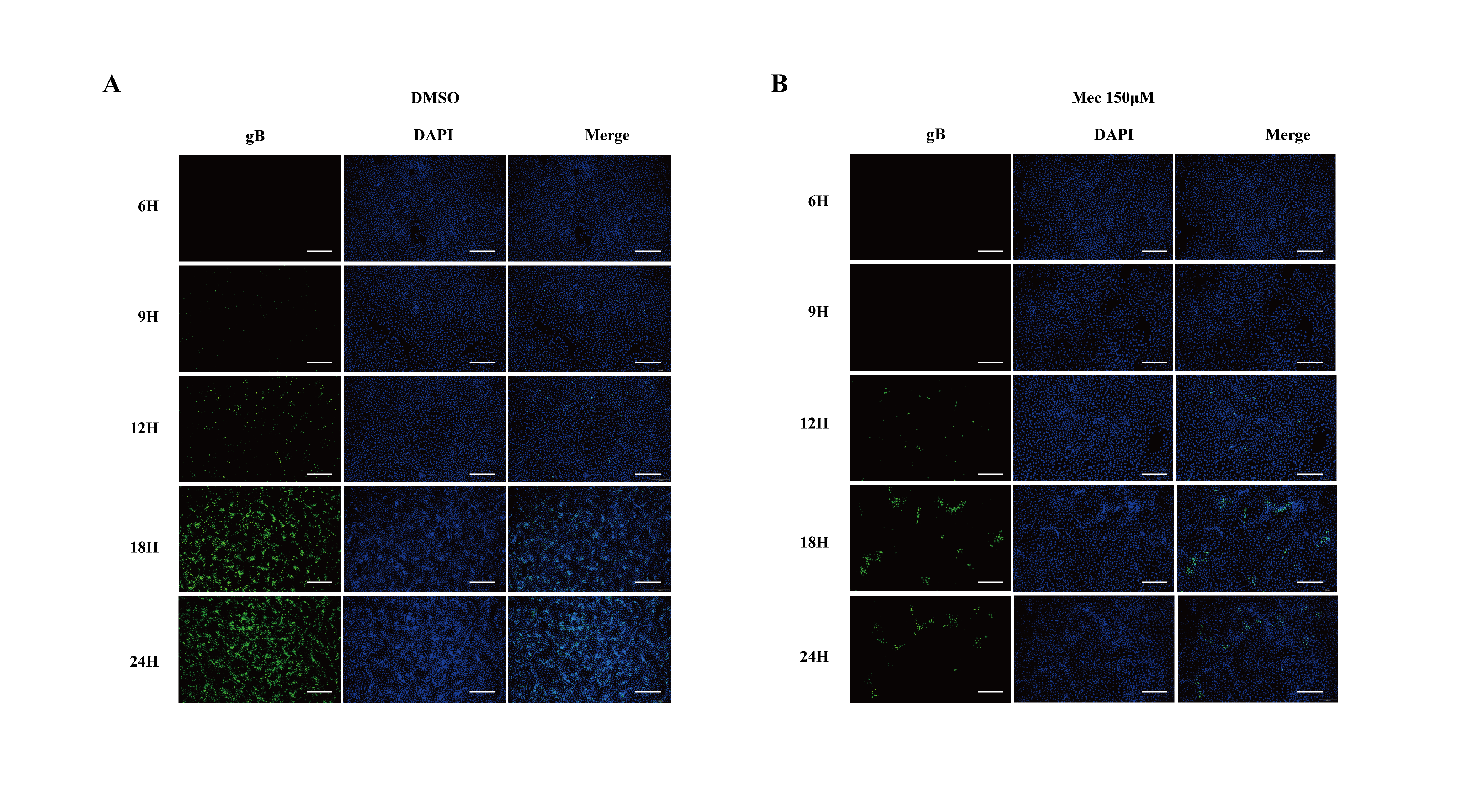

Supplement: Supplementary Figure S1 — Meclizine inhibited cell-to-cell spreading step during PRV-V replication. Scale bars = 200 μm. Typical figures were presented from three independent experiments. [file Image_1.tif]
